# Parents, Peers, and Musical Play: Integrated Parent-Child Music Class Program Supports Community Participation and Well-Being for Families of Children With and Without Autism Spectrum Disorder

**DOI:** 10.3389/fpsyg.2020.555717

**Published:** 2020-10-30

**Authors:** Miriam D. Lense, Sara Beck, Christina Liu, Rita Pfeiffer, Nicole Diaz, Megan Lynch, Nia Goodman, Adam Summers, Marisa H. Fisher

**Affiliations:** ^1^Vanderbilt University Medical Center, Nashville, TN, United States; ^2^Vanderbilt University, Nashville, TN, United States; ^3^Randolph College, Lynchburg, VA, United States; ^4^Michigan State University, East Lansing, MI, United States; ^5^Belmont University, Nashville, TN, United States

**Keywords:** community participation, autism spectrum disorder, integrated community experiences, music, well-being, parent training, music class

## Abstract

Opportunities for meaningful community participation may influence the development and well-being of individuals with autism spectrum disorder (ASD) and their families as well as impact how community members perceive and understand ASD. In the current study, we aimed to understand how a parent-child integrated music class program could be used to promote community participation and family well-being. Caregivers of preschoolers (2–5 years of age) with ASD and those of peer children with typical development (TD) were interviewed about their participation in a parent-child integrated music class program. Thematic analysis of interviews revealed that all caregivers viewed program participation as positive. Caregivers emphasized increasing connections within families, such as through strengthening parent-child bonds, as well as connections across families, including increased understanding of ASD and sensitivity to the experience of parenting. Many caregivers perceived the class as supporting their parenting and impacting their children’s behavior in meaningful ways. Interview themes were supported by measures of caregiver and child program receipt, including questionnaires about family music engagement throughout their time in the class program and behavioral coding of children’s engagement during music classes. Findings suggest that integrated community experiences such as parent-child music classes may impact whole family well-being, highlighting the value of integrated community participation experiences at the level of the family system. Parent-child music classes may provide a productive and accessible context for supporting integrated community experiences.

## Introduction

Community participation, or the involvement of individuals in societal activities (WHO, 2011), results from an interaction of both personal and environmental factors that support or hinder a person’s inclusion and integration in activities. Integrated community experiences play important roles in skill development and generalization for individuals with disabilities, and also affect the emotional well-being of people with disabilities and their families (King et al., 2003; Dunst et al., 2006; WHO, 2011; Askari et al., 2015). Opportunities to participate in and experience diverse activities through integrated community experiences may contribute to the development of relationships, including by affecting how other community members perceive and understand disability (King et al., 2003; WHO, 2011).

Increasing community participation is of particular relevance for individuals and families of individuals with autism spectrum disorder (ASD), a common neurodevelopmental disorder characterized by difficulties with social interaction and communication (American Psychiatric Association, 2013; Askari et al., 2015). For example, compared to their peers with typical development (TD), children with ASD have fewer friends (Bauminger and Kasari, 2000) and generally participate less in social and recreational activities (e.g., play dates, sports teams, art, or music lessons), especially during the school-aged and adolescent periods (Sanders and Morgan, 1997; Solish et al., 2010; Askari et al., 2015; Egilson et al., 2017). While rates of community participation are reported to be similar for toddler/preschool-aged children with and without ASD (Lam et al., 2010), such rates may be inflated due to the incorporation of errands (e.g., grocery shopping) when measuring community participation. Moreover, despite reports that parents of preschoolers with and without ASD are equally likely and willing to take their child to various community activities, such activities were perceived as more difficult for the parents of children with ASD and they experienced fewer positive and more negative emotions during these community activities (Lam et al., 2010). This was particularly the case for recreational activities (Lam et al., 2010).

Several variables have been related to the reduced community participation of individuals with ASD and their families. A parent’s negative emotional experiences while participating in community activities may impact participation in future community outings, creating a negative feedback loop that reduces community participation over time (Lam et al., 2010; Karst and van Hecke, 2012). Negative emotional experiences were related to parent’s reduced willingness to participate with their child in community activities even when controlling for the difficulty level of the community participation (while positive emotions were associated with increased willingness; Lam et al., 2010). Parents’ (specifically mothers’) own community participation in recreational activities also appears to be related to children’s participation though it is unclear if this is due to joint family participation or general family attitudes toward recreational activities (Orsmond et al., 2004). While parents of children with ASD have higher rates of parenting stress, depression, and anxiety, few community intervention programs directly address the needs of the parent (Dykens et al., 2014).

Negative attitudes of other community members (e.g., stigma) toward children with disabilities and their families can be another large barrier to participation in community activities (Gray, 2002; Howell and Pierson, 2010; Anaby et al., 2013). Alternatively, experiences of more positive attitudes toward children with disabilities and their families can support the likelihood of increased community participation (King et al., 2003; Bedell et al., 2011; Kramer et al., 2012; Anaby et al., 2013). Thus, positive community responses toward individuals with ASD, opportunities for caregiver participation, and increased positive caregiver emotions during community activities may be important factors for increasing community participation and well-being of children with ASD and their parents.

One way to shape accepting attitudes of a family system toward those with disabilities such as ASD and to create a supportive positive experience for parents and their children with ASD is to provide shared *parent-child* socialization experiences. For parents and children without disabilities, intergroup contact may reduce prejudice and negative attitudes toward families and children with ASD (Allport, 1954), as these shared experiences may be related to increased empathy (Pettigrew and Tropp, 2006). Indeed, research examining attitudes toward individuals with disabilities in school and work settings indicates that positive social contact with individuals with disabilities leads to more positive attitudes and acceptance by those without disabilities (Copeland et al., 2004; Scior, 2011; Yu et al., 2012). Providing the opportunity for parents and their child with TD to participate in integrated programming will ensure that both children and their parents have exposure to and engagement with individuals with disabilities, which may lead to more positive attitudes and interactions.

Attitudes about others are shaped by one’s socialization experiences (Maccoby, 1992). Research on how parents’ attitudes about disability impact children’s attitudes or behavior toward other children with disabilities is somewhat mixed and limited as it generally relies on questionnaires or interviews and not actual observed behavior with others (Rosenbaum et al., 1988; Hong et al., 2014). Still, one study found that children with TD whose parents endorsed that they would model interacting with a child with a disability themselves in a vignette study were more likely to actually interact with children with disabilities during preschool classroom free play (Okagaki et al., 1998). Parents may influence their young child’s peer interactions and relationships through modeling, teaching, or arranging opportunities for peer interaction (Dunn, 1993; Okagaki et al., 1998).

Thus, shared parent-child community integration experiences may have benefits for all groups involved. Children with ASD may develop and generalize skills, as well as form peer relationships. Parents of children with ASD may experience decreases in negative emotions and increases in well-being and parenting efficacy (similar to studies of parent-implemented interventions; McConachie and Diggle, 2007). For children with TD and their parents, integrated experiences may increase positive attitudes and acceptance of individuals with disabilities.

### Musical Play Contexts for Community Integration and Family Well-Being

Musical interaction activities are a natural part of parent-child, sibling, and peer-peer social experiences (Trehub and Gudmundsdottir, 2015; Politimou et al., 2018; Cirelli et al., 2020) and may provide a particularly appropriate context for integrated community experiences for young children with ASD that also incorporates parents. The PRESS-Play framework (Lense and Camarata, 2020) suggests that musical play activities may provide a setting that is particularly conducive to community integration and participation since musical experiences involve predictability, reinforcement, emotion regulation, shared attention, and social play. Such elements may scaffold participation of both children with ASD and their interaction partners (e.g., parents and peers). For example, as a highly routinized activity, musical games are familiar for children with and without ASD and their parents, helping all partners know what is expected of them as they participate in a shared activity with a common goal.

Indeed, music therapy has been used successfully to promote social interaction between children with ASD and their therapists (Kim et al., 2008) and their peers (Kern et al., 2007; LaGasse, 2015). Parents of young children with ASD also report that family-based music therapy supports the parent-child relationship (Thompson et al., 2014). Participation in music classes may be an ideal integrated community activity for families of children with ASD because many children with ASD have an interest in and propensity for music despite sensory difficulties (Dickie et al., 2009). Thus, musical activities may provide a positive and accessible platform for shared engagement that scaffolds integrated group participation and connection among children with ASD, peers, and parents and improves family well-being.

### The Current Study

In the current paper, we investigated if participation in a parent-child integrated music class program promotes community participation and family well-being in families of young children with and without ASD. We were particularly interested in caregivers of children with ASD and TD’s perceived positive and negative experiences with such a program, perceived impact of program participation on the family and awareness and attitudes toward ASD. Perspectives on these components could all contribute toward families’ willingness to participate in future integrated community activities and influence family well-being. We used a mixed-methods approach including interviews with caregivers, surveys, and behavior coding. Qualitative data regarding actual experiences in programs, as well as metrics of caregiver and child program receipt (Borrelli, 2011), can inform mechanisms by which behavior change may occur (Perkins et al., 2018). Such information is a first step toward understanding the feasibility and potential efficacy of musical experiences for promoting participation in integrated community activities and family well-being.

## Materials and Methods

### Participants

Participants included the parents/caregivers of 14 children with ASD (mean ± SD: 38.6 ± 11.2 months, four females) and 14 children with TD (34.1 ± 9.6 months, four females) who participated in one of six Serenade parent-child music groups offered as part of a research study. Participants were recruited *via* flyers for a parent-child integrated music program study posted at the university autism research center and clinics, local preschools, community sites (e.g., library), and on social media. Participants were eligible to participate if they were able to attend at least eight group sessions, were 18 months–5 years of age, and did not have prior experience with music therapy. Caregivers of TD children could not have specialized training/experience in working with children with special needs (e.g., no special education teacher or psychologist). Children with ASD, all had confirmed diagnoses *via* the Autism Diagnostic Observation Schedule, second edition (ADOS-2; Lord et al., 2012a,b) and clinician best estimate. ASD assessments were conducted by research-reliable clinicians at the same academic medical center prior to participating in the current study. Children with ASD had varied developmental levels with language skills [average of Mullen Scales of Early Learning (Mullen, 1995) Receptive/Expressive Language scales age equivalence scores] ranging from 7 to 41 months (average 19.5 ± 9.9 months).

For the families of the children with ASD, mothers were the primary parent attendees and interview participants for six families, fathers for two families, and grandmother for one family. For five additional families, both the mother and father frequently attended classes together; of these families, three mothers participated in the interview, one father, and one mother and father dyad. For the families of children with TD, mothers were the primary parent attendees and interview participants for 10 families, fathers for two families, and grandmother for one family. For the last family, both the mother and father typically attended classes but only the mother participated in the interview. Demographic information on parents is in Table 1.

**Table 1:** Demographic information of caregivers.

	Mother		Father	
	ASD^1^ (*n* = 13)	TD (*n* = 14)	ASD^1^ (*n* = 13)	TD (*n* = 14)
Age [years; mean (SD)]^2^	35.3 (4.5)	36.3 (4.4)	35.1 (6.6)	38.9 (5.7)
Education (# with 4-year college degree)^2^^,^^3^	6	13	7	12
Family Income (# > $70,000/year)^2^	8 ASD; 11 TD			
Race (# Caucasian)^4^	10	14	11	14
Ethnicity (# Hispanic/Latinx)	2	0	1	3

1 For two autism spectrum disorder (ASD) families, demographic information was provided for only one parent who was involved with child (one mother; one father).

2 One mother-father dyad of child with ASD declined to provide age/income/education information; *n* = 12 for these variables for the ASD groups.

3 Mothers of children with typical development (TD) were significantly more likely to have a college degree than mothers of children with ASD (Fisher’s exact test *p* = 0.026).

4 Mothers of children with TD were significantly more likely to be Caucasian than mothers of children with ASD (Fisher’s exact test *p* = 0.016).

### Procedures

#### Serenade Program

Serenade is a 10-week parent-child music class program, led by a board-certified music therapist, to provide music-based parent training and peer interaction in a musical play context following a manualized curriculum. During Serenade’s weekly class sessions, groups of 4–6 families (2–3 with child with ASD and 2–3 with child with TD) participate in joint music making activities that are designed to facilitate children’s engagement while also teaching parents behavioral strategies to promote children’s social engagement and positive behavior. Over the course of the program, families develop a “musical toolbox” of parenting strategies to support their child’s development. At the start of the program families receive support materials including audio recordings of the songs, a class social story (i.e., a short story with pictures and brief sentences that describes the class context, and expectations; Gray and Garand, 1993), and a class visual schedule. The class utilizes best practices for working with children with ASD that are applied for all children in the program (e.g., visual supports, prompting, and behavioral reinforcement).

During each week of the Serenade program, the music therapist focuses on a theme of using musical strategies toward a particular behavioral goal (Table 2). Each class session is structured in three segments. The first segment is a warm-up period during which children engage in free play while the music therapist leads the parents in a discussion of the previous week’s goals. The music therapist then introduces the theme for the current week and families participate in a ~30–40-min session of structured group music making activities (see below paragraph and Table 3). Throughout the music making activities, the music therapist provides instructions to demonstrate how the music activities connect to the session theme. Finally, families participate in a goal setting discussion and set a musical goal related to the session theme to practice at home during the week. Families receive a handout each week reviewing the session theme and associated music-based strategies. All families participate in all aspects of the class (i.e., caregivers of children with and without ASD set goals to practice at home and discuss their goal progress during the class discussion). In this way, the Serenade program incorporates aspects of community music classes, parent training, and parent support groups.

**Table 2:** Weekly themes of Serenade parent-child music class program.

1.	Using music to support social interaction
2.	Capturing your child’s attention with music
3.	Music for imitation and communicative gestures
4.	Promoting speech and language skills with music
5.	Music as behavioral reinforcement
6.	Music for redirection and transitions
7.	Modeling and modulating emotions with music
8.	Promoting turn-taking and making choices through music
9.	Music for pretend play and imagination
10.	Music for peer interactions and games

**Table 3:** Serenade class activities and examples of variations on song activities for session themes.

Session activity and songs	Session 3. Music for imitation and communicative gestures	Session 7. Modeling and modulating emotions with music
Check-in and discussion of at-home practice and goal from prior session. Introduce theme for the current session.		
Music-making activities:		
Greeting routine: Hello song (original)	Wave hello	Model smiling. As appropriate to child’s developmental level, label child’s emotion or ask child how they are feeling.
Small body movements: Rum Sum Sum (traditional)	Imitate hand gestures used in the song	Modulate the tempo, volume, and energy of the song and connect to emotional states.
Large body movements: We are the Dinosaurs (The Laurie Berkener band)	Imitate large body movements and song-associated gestures	Contrast verses of song for energetic marching movements vs. resting movements.
Turn-taking/choices: Flower Shop song (traditional; the type of shop and associated props change throughout sessions)	Point to a choice board to choose a flower; Imitate gestures with flower prop (e.g., smell flower)	Choose musical instruments during song; Play musical instruments in different ways (loud/fast vs. quiet/slow)
Movement/emotions: Happy and you know it (traditional)	Imitate body movements (traditional song movements and new movements)	Practice emotions and coping strategies through song lyrics and movements (e.g., happy → clap hands; sad → get hug)
Book + Song (varied): Examples include Brown Bear by Bill Martin Jr.; Octopus (Slippery Fish) by Charlotte Diamond	Point to pictures in book. Point to classmates to choose whose turn it is	Engage in movements and emotional facial expressions appropriate to song lyrics
Calming routine: Lullaby (varied). Examples include familiar nursery rhymes, original songs, and lullaby versions of popular songs	Incorporate gestures in song (e.g., hand movements to *Twinkle Little Star*)	Soothing lullaby routine (e.g., singing + rocking child) to a lullaby version of a popular song (demonstrating how can modulate any song to be a calming lullaby)
Goodbye routine: Goodbye song (original)	Wave goodbye	Model smiling. As appropriate to child’s developmental level, label child’s emotion or ask child how they are feeling.
Review session theme and provide handout to parents about theme. Parents set individual goal related to theme for at home practice.		

Each class session follows a similar musical routine using a limited set of songs with small variations that explicitly connect the musical activities to the session theme (Table 3). The music making portion encompasses eight types of songs/activities to promote the development of different social interaction skills such as attention to others, imitation, turn-taking, and emotion modulation. Song activities provide opportunities for modeling, imitation, and reciprocal interaction among the teacher, peers, and parents including through singing, communicative gestures (e.g., waving), small body movements (e.g., clapping and tapping hands), large body movements (e.g., marching and jumping), musical instrument and toy play, and pretend play. Over successive classes, variations in the songs are introduced including in the pacing, type of movements, and incorporation of props. Through these song variations and the music therapist’s direct instruction, parents learn to apply specific strategies (e.g., following their child’s leads, animacy, and positive reinforcement) to target specific goals through song activities. For example, during Theme 3 (“Music for Imitation and Communicative Gestures”), the music therapist embeds parent didactics on contingent imitation, modeling, and prompting into the song activities and families practice these skills as they complete the music activities during the session (Table 3). Parents actively participate in the activities with their children, providing support at the level appropriate to their child (e.g., hand-over-hand, gestural, and verbal prompts).

The board-certified music therapist was supported by two trained Research Assistants (RAs) who provided behavioral or technical support (e.g., to assist with recording equipment or supplies) throughout the session. RAs ranged from undergraduate to postdoctoral trainees. All study personnel were trained in the Serenade curriculum and in working with children with ASD. Fidelity ratings completed by the music therapist and an RA after each session ensured high fidelity to the program curriculum (>98% by both music therapist and RA). The Serenade program was developed for the purposes of this research project and was provided to participants free of charge. Families also received monetary compensation ($5/class attended; up to $50) and a small musical toy (egg shakers) for participating in the research. Serenade sessions occurred at a room at the university. The study was approved by the university IRB, and all parents/guardians provided written consent.

### Measures

#### Evaluation Survey and Interview

At the end of the Serenade program, a caregiver from each family completed a 14-item program evaluation survey regarding their experience and community music plans using a five-point Likert scale (Strongly Disagree to Strongly Agree ratings; survey questions in Table 4). Caregivers also participated in an individual ~15-min semi-structured interview about their experience in the program (one mother/father dyad completed the interview together). Interviews occurred in a room at the university (23 families) or *via* telephone (two families; due to scheduling). The aim of the interview was to understand caregivers’ impressions of and attitudes about participating in the integrated Serenade program. Caregivers were asked to reflect on their general impressions of the program (e.g., “What was your general impression of the music class program?”), perceived impact of the program on their family (e.g., “What changes, if any, have you or your family experienced over the course of the program?”), and if the program impacted their thoughts about parenting, autism, and their own child (e.g., “Has participation in the program influenced your approach to parenting in any way?”; “Can you reflect if the program impacted your awareness or understanding of autism?”; and “Has participation in the program impacted how you think about your own child’s strengths and weaknesses?”). Based on responses, interviewers asked follow-up questions for the caregivers to provide specific examples. Interviews were recorded for off-line transcription and coding. Three families (two ASD, one TD) were unable to complete interviews due to scheduling but did complete other study measures.

**Table 4:** Mean (SD) of caregiver ratings on the Program Evaluation Survey for the ASD and TD groups.

S. No.	Item	ASD (*n* = 14)	TD (*n* = 14)
1.	I enjoyed participating in the music classes.	4.9 (0.3)	4.7 (0.5)
2.	My child enjoyed participating in the music classes.	4.8 (0.4)	4.7 (0.5)
3.	I know more about how I can engage in music activities with my child.	4.8 (0.4)	4.5 (0.5)
4.	I use music in an intentional way to engage and interact with my child.	4.6 (0.5)	4.5 (0.5)
5.	I use music in an intentional way to support my child in everyday activities, transitions, and routines.	4.4 (0.7)	4.6 (0.5)
6.	I have noticed improvements in my child’s engagement in musical activities.	4.4 (0.6)	4.1 (0.7)
7.	I have noticed improvements in my child’s social behaviors.	4.4 (0.6)	3.9 (1.0)
8.	I have noticed improvements in my child’s communication behaviors.	4.2 (0.7)	3.8 (0.9)
9.	I have noticed improvements in my child’s participation in daily routines.	4.0 (0.8)	4.0 (0.7)
10.	The handouts and visual supports were helpful.	4.3 (0.7)	4.2 (0.8)
11.	The music materials (recordings) were helpful.	4.8 (0.4)	4.6 (0.5)
12.	I will bring my child to musical activities in the community.	4.4 (0.9)	4.7 (0.5)
13.	I would recommend Serenade parent-child music classes to other families.	4.9 (0.3)	4.8 (0.4)
14.	If a different Serenade parent-child music class series was offered, I would be interested in participating.	4.7 (0.9)	4.7 (0.6)

Likert scale: 1 = Strongly disagree; 2 = Disagree; 3 = Neither agree nor disagree; 4 = Agree; and 5 = Strongly Agree.

#### Weekly Home Engagement Questionnaire

Throughout their participation in the program, a caregiver from each family completed a weekly questionnaire reporting on their family’s music engagement at home. Parents reported the number of days they worked on their specific goal and the amount of time/day spent in social musical activities (i.e., musical activities with another person), as well as completed a yes/no checklist of 10 reasons why they may have used musical play that week (potential reasons enumerated in Table 5).

**Table 5:** Average (SD) proportion of weeks families of children with ASD and TD engaged in musical activities for a given reason at home during their 10-weeks of participation in the Serenade program.

Reason	ASD (*n* = 14)	TD (*n* = 14)
Social games	82.6% (16.2%)	77.0% (19.8%)
Soothe child^*^	68.6% (28.2%)	33.6% (25.3%)
Transitions (switching to a new activity)	57.6% (27.5%)	63.1% (26.8%)
Distraction	57.0% (28.8%)	56.2% (27.9%)
Attract attention^*^	48.7% (34.5%)	17.6% (24.6%)
Communication^*^	66.1% (25.9%)	33.6% (24.8%)
Nighttime routine	50.9% (40.2%)	31.3% (32.4%)
Daily living routines (e.g., getting dressed)	50.6% (34.9%)	66.1% (25.5%)
Pretend/Imaginative play	29.8% (27.6%)	32.6% (27.3%)
Positive reinforcement	22.5% (17.2%)	29.7% (26.5%)

* Significantly greater in ASD than TD.

#### Children’s Class Engagement

Children’s engagement in class was coded using a 5-s momentary coding scheme from videos of the first, middle, and last class session for each child (typically the first, fifth, and tenth sessions but the next closest session was coded in the case of absences). For each interval, children’s behavior was scored hierarchically as actively engaged [overt movement activity (e.g., singing, song-related hand gestures) while attending to class members/activities], facilitated engaged (parent or RA physically prompting child’s movement while child attending to class members/activities), passively engaged (no overt movement but attending to class members/activities), unengaged (child not attending to class including off-task behavior), and disruptive (e.g., screaming). Uncodable intervals (e.g., child not visible on video) were also noted. Attention was determined based on children’s activity-related movement and/or gaze direction as appropriate to the code. Engagement was coded for six of the eight song activities. Engagement was not coded during the book singing activity because changes in the book and book-related activities across sessions could have biased engagement coding (e.g., familiarity of text/lyrics and differing amounts of movements associated with different books). Engagement was also not coded during the lullaby activity since children were not expected to sing or move for the lullaby. Proportion of time in each engagement state was calculated as number of intervals in each engagement state (active, facilitated, passive, unengaged, and disruptive) divided by number of total codable intervals across the six coded song activities. Videos were coded by three RAs. Around 13% of participants were coded by a second RA for reliability (ICC’s: active: 0.936; facilitated: 0.947; passive: 0.836; unengaged: 0.875; and disruptive: 0.723).

### Data Analysis

#### Interview Coding and Analysis

Coding drew upon a thematic analysis approach (Braun and Clarke, 2006) based on the overarching research goal of understanding elements of the parents’ experiences that relate to community participation, integration, and family well-being. Such coding is an iterative approach that involves re-reviewing transcripts as codes emerge. First, all interview transcripts were reviewed to gain familiarity with their content. One researcher then reviewed the transcripts line-by-line and labeled all relevant lines with specific codes as appropriate to the research goal. The initial coder and a second researcher then organized all codes and examined codes for the sample as a whole as well as by diagnostic group to search for codes that grouped together. Codes were then collated into five relevant themes. For example, initial coding identified specific behavior strategies that families employed; these were then combined under a general theme of “Parenting Strategies.” The two researchers then reviewed these themes against the transcripts and independently coded whether each theme was represented in each interview transcript. In cases of disagreement (8% of the 125 codes), the researchers discussed and refined the themes and came to consensus. Themes were named and reviewed with regard to their relationship to community integration and family well-being throughout the process as appropriate to the iterative process.

#### Home Engagement Questionnaire, Program Evaluation Survey, and Child Class Engagement Coding Analyses

Descriptive measures of caregiver and child program receipt were examined. We summarized measures of at-home music engagement as grand median of days families practiced their goal per week; grand median of time families spent in social musical activities per day [medians were used for these metrics because these items were scored from binned numbers of days (e.g., 0, 1–2, and 3–4 days)]; mean number of reasons why families used music each week, and mean percent of weeks they used music for specific purposes. We also provide individual item ratings on the parent evaluation survey. For children’s class engagement, we focused analysis on children’s active engagement during class (i.e., child singing or completing song-associated movements while attending to the class members/activities). We conducted repeated measures ANOVA to examine differences in children’s active class engagement over time by diagnostic group. Given the small sample size, we consider any statistical findings to be exploratory and we present them here to complement the parent interview information.

## Results

The five major themes that emerged from the interviews were: (1) positive experience of class participation, (2) awareness and empathy, (3) family connections, (4) parenting strategies and skill learning, and (5) perceived changes in child behavior. Below we discuss these themes in more detail including specific examples and similarities and differences in responses from families of children with and without ASD. As relevant, descriptive information of program receipt by caregivers and children (Home Engagement Questionnaire, Program Evaluation Survey, and behavioral coding of children’s class engagement), is provided to support the findings from the caregiver interviews.

### Theme 1: Positive Experience of Class Participation

All caregivers (25 of 25 interviews) reported on positive aspects of class participation (i.e., their experience during the class itself). Within this theme, two subthemes emerged: (1) the class was fun and supportive and (2) community participation opportunities.

#### The Class Was Fun and Supportive

Caregivers of both children with and without ASD particularly commented on the class being fun, on the supportive environment that met all children at their level, and on the use of routines that helped children become familiar with the activities each week. For examples, caregivers stated:

She [child with ASD] definitely looked forward to it….that’s the most positive thing for us [ASD caregiver]

And it was easy, that was the thing. You made it very easy ….everything’s challenging,….but it [the music class] was very autistic-friendly, it was music-friendly, it was family friendly [ASD caregiver]

I think it was a place where all of the children grew to be very comfortable and looked forward to coming to, which speaks volumes about how it was handled there. Even when they weren’t doing what was asked of them, they were responded to positively, which was good for everybody. [TD caregiver]

These qualitative findings were also corroborated by program evaluation survey data where caregivers reported that both caregivers and children enjoyed the class experience (Table 4).

#### Community Participation Opportunities

Creating an environment that is supportive and enjoyable for all families may be reinforcing and increase families’ willingness to participate in this and other integrated community experiences. This is evident from responses on the evaluation survey, in which caregivers of children with and without ASD expressed interest in participating in additional music classes and in attending music activities in the community (Table 4). The caregivers also indicated they would recommend the integrated music program to others (Table 4). Comments suggested that for caregivers of children with ASD, the integrated music class may have made families more confident about community participation more broadly. For example, one caregiver stated:

Music class has let me see that there’s nothing we can’t do….yes – we might have to tweak it, yes – we might have do a little extra hand-holding, but there’s no reason that he can’t participate in everything that a typically developing child can participate in [ASD caregiver]

Caregivers of children with TD became more aware of considering integrated opportunities when deciding on community activities. For example, one caregiver of a child with TD stated, “I would definitely want to do [integrated community activities]. I never thought about it before.”

### Theme 2: Awareness and Empathy

A second theme directly addressed the integrated group participation aspect of the class with caregivers commenting about interactions with other families (9 of 12 ASD; 12 of 13 TD). Three sub-themes emerged as both caregivers of children with (1) TD and (2) ASD expressed sensitivity to the challenges of parenting but in different regards, and they also (3) discussed how the class impacted their children.

#### Caregivers of Children With TD

Many caregivers of children with TD spoke candidly about learning about the experiences of children with ASD and their parents and reported increased empathy due to this interaction. For example, one caregiver stated, “I don’t have much experience with kids with ASD….I feel like it may have increased my sensitivity in a good way.” [TD caregiver] Another caregiver of a child with TD commented, “It makes me realize I’m not going to judge anyone anymore because you don’t know what their kid is dealing with or the parent’s dealing with.”

Many caregivers of children with TD expressed a greater awareness of the variability of presentations in ASD following exposure to children at different levels of functioning and communication. For example:

I had very little exposure previously. Never really that close of interaction with kids with autism. My wife’s cousin is on the spectrum but not as severe as the kids in the class. [TD caregiver]

I think my mouth sort of dropped when mom of [child with ASD] was like ‘oh he’s in this therapy, this therapy, this therapy.’ I never would have picked up on that. [TD caregiver]

Some TD caregivers also spoke about increased awareness of opportunities to support families of children with ASD through the process of having actual interaction experiences. For example, a caregiver commented,

being in an inclusive [integrated] class like that gives you an opportunity to see how you could [be] helpful and to see what they’re dealing with…. I think it takes a little bit of the fear out of it and it becomes a lot more realistic…where just being able to witness it and experience it helps you figure out how you can help somebody else….you don’t really know until you’re around somebody. [TD caregiver]

And the little pieces but wanting to celebrate, because I feel like I saw some of the progress with the kids in their involvement [in the music class]. I knew that the parents were working really hard at home. The celebratory difference and [to] see [children with ASD] respond differently from the first class to the last. [TD caregiver]

For the caregivers of children with TD, the shared experiences of group music making (which might require various facilitating or prompting techniques) and working on musical goals to support their children’s behavior seemed to lead to an overarching identification as parents working to engage with their children. For example, one caregiver stated, “It was nice because I got to see parents struggling with the same things I was struggling with…we’re all in this together, trying to raise good kids” [TD caregiver]. Additionally, a caregiver of a child with TD who joined a commercially available class following participation in Serenade felt that in the integrated program, “there was just a lot more understanding and working together to help each person.” Another caregiver summarized the experience as, “Being in a group I guess that is accepting of you and your child reinforces your ability as a parent” [TD caregiver].

#### Caregivers of Children With ASD

In contrast, caregivers of children with ASD generally commented that raising a child with ASD had made them more sensitive to and empathetic about parenting and that they recognized this dynamic during the music class. For example, one caregiver of a child with ASD stated, “I’d say I considered [parenting] in the course of the class… I’m much more understanding in that situation, and I noticed that in the music class.” Another said,

Having a child with autism has in general changed how I perceive other parents’ behaviors…it just kind of reaffirmed that I’m much more empathetic or understanding of how parents are interacting with their children [ASD caregiver]

#### Children With and Without ASD

Caregivers of children with TD and ASD both valued their child having peer interactions during the class. Some caregivers of children with ASD liked having a place where their child “is not going to stand out” and were able to interact with other children with ASD as well as learn from the peers with TD. For example, one caregiver stated, “[that] was a really good part for her, to see what the other kids were doing” [ASD caregiver].

Caregivers of children with TD also valued their child having an integrated experience. One caregiver of a child with TD commented that the best part was how “the other kids were really trying to help [child with ASD].” Another reported:

Having a mixed group – some people see it as a disadvantage, and I see it as a positive thing – kids with ASD and typical kids….I think it’s something good for my child to be exposed to differences and to be able to accept those differences. [TD caregiver]

### Theme 3: Family Connections

Many caregivers related that the class facilitated a sense of bonding or connection with their child (8 of 12 ASD; 6 of 13 TD). Two sub-themes emerged related to (1) parent-child connections and (2) musical activities with other family members.

#### Parent-Child Connections

Participating in the class helped caregivers to develop a shared activity with their child that they could use to intentionally engage with each other during class and through at-home practice.

It was nice to have a weekly hour where it was dedicated for very intentional play with [son], and I’ve appreciated that more where you can sit down and play and engage with him more rather than do a million things around the house while he’s playing. Emphasizing the importance of intentional play with your child is helpful, helps you understand the importance of it. [TD caregiver]

The family connections may have related to Theme 4 of parenting strategies and Theme 5 of perceived changes in child behavior (below) as caregivers sometimes reported more confidence in interacting with their child. For example, one caregiver stated, “It’s [child’s] way of letting us come into his world and…share part of that” [ASD caregiver]. Several mothers of children with ASD indicated that the program provided bonding and learning opportunities for their spouses since the fathers typically were not involved in their child’s therapies. One mother commented that with the music, the “[father] was more willing to be close to [child]… That was helpful as a family.” Another mother stated, “For [husband], it was huge because it was time he had to spend with [son]…[Husband] feels more confident” [ASD caregiver].

#### Musical Activities With Other Family Members

Caregivers also reported sharing the musical activities with non-attending family members like siblings or grandparents leading to additional family interaction opportunities.

It was family friendly…it’s something that somebody easily – his dad, his grandpa, everybody that even wasn’t there in the class they picked up on very easily. [ASD caregiver]

[Child with ASD] kind of interacts with [younger TD sister] a little bit when we do the dancing or the songs…[sister says]‘come on brothers’ and then she’ll try to sing or to dance with him. [ASD caregiver]

These interview responses were consistent with caregivers’ reports on their weekly Home Engagement Questionnaires, whereby families’ program engagement extended to their at-home musical interactions with their child. On the weekly Home Engagement Questionnaire, caregivers reported that their child engaged in social musical activities (e.g., with parent or other family members) approximately 20–30 min per day in the ASD group and 10–20 min per day in the TD group.

### Theme 4: Parenting Strategies and Skill Learning

An additional major theme from the interviews was caregivers learning and applying specific strategies for supporting their child’s behavior due to class participation (24 of 25 interviews). Two subthemes emerged, including (1) music-based parenting strategies and (2) generalization of parenting strategies.

#### Music-Based Parenting Strategies

Compared to many commercially available programs that focus on musical development, Serenade is explicit about caregivers learning and practicing different parenting skills through weekly musical goal assignments for at-home practice, handouts about strategies, modeling techniques in class, and discussion and feedback during class. The interviews demonstrated that caregivers of children with TD and ASD were receptive to learning and applying music-based strategies for supporting their child’s behavior. Many of the strategies endorsed by caregivers during the interviews involved their incorporation of music for specific purposes typically through the use of parent song.

It just wasn’t about music, it was about transitioning, about cleaning up, about bedtime, you know, routines….stuff that for a typical child might have a few challenges, but for an autistic child, a simple task like sitting down to dinner can be really rough [ASD caregiver]

You can extract things from any song and turn into a game, it really gets them moving and motivating instead of a battle of will, which sometimes it can be. [TD caregiver]

I feel like it’s been good for me to think a little more creatively how I can motivate him using music or just redirecting him with music, or as a reward. I wouldn’t have thought about using music specifically. [TD caregiver]

These interview findings are consistent with caregivers’ responses on the weekly Home Engagement Questionnaire. Throughout their time in the program, caregivers reported working on the specific goal they set 4–5 days per week in the ASD group and 3–4 days per week in the TD group. Moreover, caregivers reported using musical activities for an average of 5.7 (2.0; ASD group) and 4.9 (1.6; TD group) reasons each week. Caregivers of children with ASD and TD reported frequently using music for social engagement, daily living routines (e.g., as part of brushing teeth or getting dressed), transitions (changing from one activity to another), and distraction (e.g., singing in car). Compared to caregivers of children with TD, caregivers of children with ASD more frequently used musical activities for soothing purposes (*t*_26_ = 3.78, *p* < 0.001), communication goals (*t*_26_ = 3.38, *p* = 0.002), and to attract their child’s attention (*t*_26_ = 2.77, *p* = 0.01; Table 5). On the program evaluation survey (Table 4), caregivers of children with ASD and children with TD reported that they knew more about how to engage in musical activities and used music in intentional ways to interact with and support their child.

#### Generalization of Parenting Strategies

One of the rationales of the Serenade curriculum is that the music-based parenting strategies are specific and concrete, but that over time, caregivers expand beyond the music-based activities to utilize the techniques more broadly in their interactions. Thus, the curriculum ties the music-based strategies to broader principles for supporting children’s behavior through the use of evidence-based supports and strategies such as visuals, social stories, prompting, and behavioral reinforcement (Wong et al., 2015). These techniques are not unique to a music class, but many caregivers of children with TD and ASD reported becoming familiar with these strategies through them being modeled and discussed in the music class and they saw how they could use them in other aspects of their daily lives.

I think a huge thing was, [music class] was our first experience with visual schedules, which was huge for him. We use it daily, preparing him for new preschool. We are showing him pictures where he is going to meet and what he is going to do. [ASD caregiver]

The strategies that were used in the class were helpful for parenting period, whether autism or typically developing. [TD caregiver]

### Theme 5: Perceived Changes in Child Behavior

Changes in the child’s behavior were another common theme through the interviews, though this was more frequently mentioned by caregivers of children with ASD (10 of 12 interviews) than caregivers of children with TD (6 of 13 interviews), and it is important to emphasize that changes are based upon caregivers’ perceptions. Changes in behavior typically related to musical behaviors or social communication or play skills that emerged during song activities (both at home and in class). A parent of a child with ASD commented, “He speaks more, I mean he interacts and waves more. Gives more eye contact, especially when you start singing the song. He recognizes it and focuses.” A parent of a child with TD mentioned, “At home he’s always “playing” music class. He’s taking his guitar, taking out the ukulele and say hello to everybody and even the stuffed toys. We just feel like he just blossomed too.”

As this was a new experience for the families and since caregivers were introduced to parenting skills during the program, it is possible that perceived changes were due to the novel experience and changes in parental behavior or parent-child interactions. Additionally, for many caregivers of children with ASD, this was the first time they saw their child around other children. Even for those attending preschool, caregivers did not necessarily get to see their child interact with others in that setting and thus, they were able to see many skills they did not know the child had. For example, one caregiver of a child with ASD commented, “With some of the interactive pieces in the class…seeing her do things, things I didn’t realize she was capable of.” Another mentioned, “I’ve never seen him…repeat gestures like that, so to just all the sudden do it was just incredible.”

These caregiver impressions are supported by the behavior coding of children’s engagement during class sessions (Table 6). Children with ASD and children with TD were primarily passively engaged in the class (i.e., attending to class members/activities but not using overt motor movements/singing). However, both children with ASD and TD increased their active engagement (attending while also actively singing/song-associated movements) in the class over time [*F*_2,52_ = 14.109, *p* < 0.001, partial η^2^ = 0.352; There was no effect of diagnosis (*F*_1,26_ = 2.895, *p* = 0.101, partial η^2^ = 0.100) or time × diagnosis interaction (*F*_2,52_ = 0.594, *p* = 0.556, partial η^2^ = 0.022)]. Thus, children generally attended to class activities and, over time, increasingly completed song-associated movements perhaps due to increased familiarity and comfort with class activities. The interviews suggest that caregivers were attuned to their children’s increased engagement in the class activities.

**Table 6:** Proportion of time in each engagement state across an early, middle, and late class session for the ASD and TD groups [mean (SD)].

	ASD (*n* = 14)			TD (*n* = 14)		
	Early	Middle	Late	Early	Middle	Late
Active	0.11 (0.10)	0.18 (0.13)	0.22 (0.12)	0.18 (0.15)	0.27 (0.15)	0.28 (0.14)
Facilitated	0.12 (0.11)	0.12 (0.09)	0.09 (0.07)	0.15 (0.14)	0.07 (0.07)	0.05 (0.07)
Passive	0.65 (0.11)	0.61 (0.12)	0.61 (0.17)	0.64 (0.16)	0.61 (0.13)	0.65 (0.13)
Unengaged	0.12 (0.10)	0.09 (0.14)	0.06 (0.10)	0.02 (0.12)	0.03 (0.04)	0.01 (0.02)
Disruptive	0.01 (0.02)	0.01 (0.01)	0.03 (0.06)	0.0 (0.0)	0.00 (0.01)	0.00 (0.01)

## Discussion

In the current study, we examined family experiences of participation in Serenade, a 10-week integrated parent-child music class program that is designed to promote social engagement and positive behavior in children with and without ASD through the use of parent training and peer integration in a musical play context. Five common themes emerged from interviews with families of children with and without ASD who participated in Serenade. These themes included (1) positive experiences and community participation, (2) awareness and empathy, (3) family connections, (4) parenting skills, and (5) children’s behavior. These findings from families of children with and without ASD suggest that integrated parent-child music classes may be a viable forum for increasing community participation and well-being of families of children with ASD, and that the integrated context may benefit both families of children with ASD and families of children with TD.

This study is unique because Serenade provides integrated community participation for children with ASD and their parents. A group music-based program was selected as the forum for participation because of the ecological validity of parent-child music classes and the interest in music displayed by children with and without ASD. Caregiver’s feedback about their experience in the program directly and/or indirectly aligned with the principles of the PRESS-Play framework (Lense and Camarata, 2020): the musical activities provided positive (reinforcing) opportunities for parents and children to share attention with each other while engaging in natural social play activities. Children increased active engagement in music session activities and parents learned to employ music-based parenting strategies, both likely supported by the predictability of the musical routines. Caregivers reported using music at home with their child for a variety of reasons including social play routines, emotion regulation, and to support specific behaviors. A growing body of qualitative and quantitative literature examines group musical experiences as a medium for targeting community engagement, participation, and well-being for the general public as well as clinical populations (Pearce et al., 2015; Lee et al., 2016; Perkins et al., 2016, 2018; Weinberg and Joseph, 2017). Integrated community participation opportunities such as group music-making activities may impact well-being through creating positive emotional experiences for participants and decreasing negative attitudes or stigma from other community members.

Feedback from families of children with TD and ASD in the current study was generally consistent with that provided by adults with TD and adults with medical or mental health difficulties participating in other types of group music making (e.g., community choirs or drum circles). For example, prior studies found that group musical activities were associated with increased well-being, social connection and meaning (Williams et al., 2012; Pearce et al., 2015; Lee et al., 2016; Perkins et al., 2016; Weinberg and Joseph, 2017), feelings of accomplishment, and increases in positive and decreases in negative emotions (Lee et al., 2016; Fancourt and Perkins, 2018; Perkins et al., 2018). Such constructs have also recently been examined within parent-child group music experiences. In focus groups with women with vulnerability to postnatal depression participating in a parent-infant music class, mothers reported increased positive emotions (for both parent and baby), feelings of accomplishment (learning new skills), and enjoyment in group participation (Perkins et al., 2018). Such experiential data are corroborated by ratings of increased parent-infant closeness, decreases in negative emotions, and decreases in cortisol in mothers participating in parent-infant music class vs. non-music play classes (Fancourt and Perkins, 2018). The current study extends these findings to both families of children with TD and ASD participating in an integrated music class.

Examining community participation through parent-child experiences is consistent with the increasing recognition of the need to consider parent and family outcomes such as emotional well-being, parenting efficacy and stress, and the parent-child relationship for families of children with ASD (Karst and van Hecke, 2012). The themes that emerged from parent interviews of both families of children with TD and ASD in the current study suggest that community participation in an integrated parent-child music class supports family well-being in alignment with frameworks such as the PERMA model (Seligman, 2011). According to the PERMA model (Seligman, 2011), well-being involves positive emotion, engagement, positive relationships, meaning, and accomplishment.

In regard to positive emotion, families frequently focused on the “fun” experience of class for both parent and child. Such positive experiences are important in order to reinforce the experience of integrated community participation for parents of children with ASD and TD. In regard to engagement, some families of children with ASD mentioned that they would not have signed up for a general parent-child music class but that the supports in the Serenade program, and the knowledge that it was for children functioning across a spectrum of abilities, made them comfortable in engaging with this new experience. At the same time, families of children with TD also appreciated the classroom supports that helped all children (including children with TD) engage in the program, suggesting that community programs should incorporate such supports to facilitate engagement of all children and families. Engagement was also reflected *via* parents’ reports of home practice of parent-child music activities and behavior coding demonstrating that children generally attended to class members/activities and increased their overt singing/song-associated movement activity over time.

Two of the themes reflected relationships including relationships within families and between families in a class. There were a relatively high proportion of fathers who attended the program, particularly for families of children with ASD. This was likely in part due to scheduling (four of the six classes met on weekends) but may also speak to a potential opportunity for community music programs to serve as a vehicle for father-child interaction. Caregiver responses addressed the accessibility of program activities such that the music activities and strategies could be shared with other (non-attending) family members, consistent with the ubiquity of musical activities in parent-child and sibling interactions (Politimou et al., 2018; Cirelli et al., 2020). In a prior focus group of four families of children with ASD participating in a group music therapy class, the parents also commented on the value of having a whole family activity (Allgood, 2005). Parents of children with ASD report that participating in individual family-centered music therapy supports their parent-child relationship (Thompson, 2014; Thompson et al., 2014).

The development of relationships across families was also an integral part of the experience and appears to have developed through the shared experiences of both music making and working on music-based behavioral goals. Such intergroup relationships may be important for reducing negative attitudes (Allport, 1954), which are a barrier to participation (Gray, 2002; Howell and Pierson, 2010; Anaby et al., 2013). These relationships may also have contributed to a sense of meaning as families spoke about empathy for their own and others’ children as well as the experiences of parents. Anecdotally, class RAs observed that when children demonstrated specific skills in a session for the first time (e.g., waving during a hello song), this was frequently recognized and socially reinforced by all class parents (e.g., clapping, cheering, and smiling at the child and his/her caregiver).

Relatedly, families reported a sense of accomplishment in regard to their child demonstrating specific skills in the class or in using skills at home. These included musical and non-musical skills and were mentioned by caregivers of both children with TD and ASD. These caregiver impressions were supported by behavior coding indicating that children increased their active engagement in class activities over time. While any objective effects of Serenade participation on children’s musical or non-musical skills outside of the music class is beyond the scope of this paper, any changes would likely relate to family practice at home and not merely attending a once-per-week program. Therefore, it is noteworthy that parents embraced the music-based parenting strategies and expressed accomplishment in their own parenting skills based on the techniques they practiced in the program. Prior studies have reported increased social behaviors, such as joint attention and turn-taking in young children with ASD during individual music therapy with a clinician (Kim et al., 2008) and in school-aged children with ASD during a music therapy social skills group (LaGasse, 2015) while the impact of individual music therapy on social skills outside of therapy sessions is mixed (Bieleninik et al., 2017; Sharda et al., 2019). Future studies could further investigate how specific parent strategies within music classes (e.g., facilitation/prompting behaviors) and at home contribute to children’s skills both during and outside of music sessions.

The current interviews, reported by parents about their experience, may also shed light on potential impacts, as well as underlying behavioral processes, of music therapy for children with ASD. While there has long been an interest in using music for social skills in ASD, music therapy is currently considered to have an emerging evidence basis by the National Autism Center’s (USA) National Standards Project (National Autism Center, 2015). Recognizing that there are structural and conceptual differences between specific individualized music therapy approaches and the current parent-child integrated music class, some areas of common focus may be worthwhile for future study. In particular, the current findings support the use of music as a platform for both parent-child and peer interactions and suggest that music therapy studies may want to consider outcomes for parents and peers and not just children with ASD. For example, parents of young children with disabilities including ASD reported improvements in parent mental health following participation in a parent-child music therapy program on a short questionnaire (Nicholson et al., 2008; Williams et al., 2012). Individual music therapy for school-aged children with ASD was associated with improvements in family quality of life (Sharda et al., 2018). School-age children with TD in an integrated (vs. non-integrated) school music program showed improvements in prosocial emotions in response to a hypothetical bullying scenario of a child with ASD (Cook et al., 2018).

Limitations of the current study include the small sample size and relatively high earnings of participating families. Maternal education in particular was higher in families of children with TD than families of children with ASD. Future research will need to include peer families from a wider educational/economic spectrum to examine the generalizability of the experiences of families of children with TD. At the same time, the participating families generally did not have prior experience with children with ASD (e.g., no special educators or therapists). As we only studied one program, we cannot comment on if results are specific to the Serenade program or if other programs – including those that isolated the effects of the music or parent education components – would have similar findings. Nevertheless, the Serenade program’s combination of parent-child music class and parent education appealed to both families of children with TD and ASD, which is integral to building an integrated community experience. Indeed, both families of children with TD and ASD requested to continue the program following the research study, suggesting that such experiences promote continued integrated community participation. Future studies can build upon these results by comparing different types of integrated programming and examining long-term attitudes toward and experiences with community participation. The current study suggests it is beneficial to consider experiences that incorporate integration of both parents and children in order to support community participation and family well-being and that parent-child music classes may provide an effective forum for working toward this goal.

## Data Availability Statement

The raw data supporting the conclusions of this article will be made available by the authors, without undue reservation.

## Ethics Statement

The studies involving human participants were reviewed and approved by Vanderbilt University Institutional Review Board. Written informed consent to participate in this study was provided by the participants’ legal guardian/next of kin.

## Author Contributions

MLe and SB conceived of the study, participated in the design, coordination, and acquisition of data, contributed to analysis, and drafted the manuscript. CL and RP participated in the study design and coordination and acquisition of data. ND participated in data measurement and contributed to analysis. MLy and NG participated in data measurement. AS participated in study design and data acquisition. MF participated in study design and drafted the manuscript. All authors contributed to the article and approved the submitted version.

### Conflict of Interest

The authors declare that the research was conducted in the absence of any commercial or financial relationships that could be construed as a potential conflict of interest.
